# circST6GALNAC6 suppresses bladder cancer metastasis by sponging miR-200a-3p to modulate the STMN1/EMT axis

**DOI:** 10.1038/s41419-021-03459-4

**Published:** 2021-02-10

**Authors:** Shuo Tan, Ye Kang, Hu Li, Hai-Qing He, Long Zheng, Shui-Qing Wu, Kai Ai, Lei Zhang, Ran Xu, Xuan-Zhi Zhang, Xiao-Kun Zhao, Xuan Zhu

**Affiliations:** 1 Department of Urology, the Second Xiangya Hospital of Central South University, Changsha, Hunan Province P R China; 2 Department of Urology, Xiangya Hospital, Central South University, Changsha, Hunan Province P R China; 3 Department of Urology, An Xiang Xian People’s Hospital, Anxiang, Hunan Province P R China

**Keywords:** Bladder cancer, Bladder cancer

## Abstract

Bladder cancer (BCa) is an aggressive malignancy because of its distant metastasis and high recurrence rate. Circular RNAs (circRNAs) exert critical regulatory functions in cancer progression. However, the expression patterns and roles of circRNAs in BCa have not been well investigated. In this study, we first screened circRNA expression profiles using a circRNA microarray of paired BCa and normal tissues, and the expression of circST6GALNAC6 was confirmed by qRT-PCR and fluorescence in situ hybridization (FISH). MTT, colony formation and Transwell assays were performed to measure cell proliferation, migration and invasion. We investigated the regulatory effect of circST6GALNAC6 on miRNA and its target genes to explore the potential regulatory mechanisms of circST6GALNAC6 by chromatin immunoprecipitation (ChIP), RNA immunoprecipitation (RIP), MS2-tagged RNA affinity purification (MS2-TRAP), immunofluorescence (IF) and dual luciferase activity assays. A nude mouse xenograft model was used to examine the functions of circST6GALNAC6/STMN1 in tumour metastasis in vivo. We found that 881 circRNAs were significantly dysregulated in BCa tissues compared to normal tissues. circST6GALNAC6(hsa_circ_0088708) was downregulated in BCa tissues and cells. Overexpression of circST6GALNAC6 effectively inhibited the cell proliferation, migration, invasion and epithelial–mesenchymal transition (EMT) in vitro and suppressed BCa metastasis in vivo. Mechanistically, we showed that the SP1 transcription factor, which binds to the circST6GALNAC6 mRNA transcript, activates circST6GALNAC6 transcription. Next, we verified that circST6GALNAC6 serves as a sponge that directly binds miR-200a-3p to regulate stathmin (STMN1) expression. Furthermore, we found that STMN1 is involved in circST6GALNAC6/miR-200a-3p axis-regulated BCa EMT and metastasis. Thus, our findings indicate an important underlying mechanism in BCa metastasis by which SP1-induced circST6GALNAC6 sponges miR-200a-3p to promote STMN1/EMT signalling. This mechanism could provide pivotal potential prognostic biomarkers and therapeutic targets for BCa.

## Introduction

Bladder cancer (BCa) is one of the most prevalent malignancies throughout the world, and millions of people die from the disease every year^1,2^. Accumulating evidence indicates that BCa represents a group of molecularly heterogeneous diseases with diverse clinical courses and therapeutic responses^3,4^. Metastases occur more frequently in muscle-invasive BCa patients and correlate with poor clinical prognosis^5^. Until now, no effective therapeutic approaches have been made available for BCa patients with tumour metastasis^6^. Therefore, a better understanding of the molecular mechanisms of BCa metastasis is necessary for the development of efficient therapeutic strategies.

Epithelial–mesenchymal transition (EMT) is a process in which epithelial cells acquire mesenchymal features by decreasing the expression of epithelial markers such as E-cadherin and increasing the expression of mesenchymal markers such as N-cadherin and vimentin^7^. It is a critical process for the invasion and metastasis of tumour cells, and many molecules are involved in this process^7^. MicroRNAs (miRNAs) are a widely acknowledged class of non-coding RNAs that suppress the expression of their targets by binding the 3′ UTRs of downstream messenger RNAs (mRNAs)^8^. The miR-200 superfamily is composed of five members, miR-200a/b/c, miR-141, and miR-429^9^. They are highly expressed in epithelial cells, and many studies indicate that the miR-200 superfamily is crucial for EMT^7,9^. For instance, previous studies have shown that miR-200a promotes BCa invasion by targeting Dicer, while miR-200b suppresses EMT and prostate cancer growth and metastasis^10,11^. miR-141 also inhibits EMT and suppresses ovarian cancer cell migration^12^. Nevertheless, the upstream regulators and whether there are additional downstream targets of the miR-200 family in BCa are poorly understood.

Circular RNAs (circRNAs) are a relatively new family of non-coding endogenous RNAs with circular configurations and stable structures^13,14^. Initially, they were considered cellular junk RNAs. However, emerging evidence suggests that their expression is highly regulated, with developmental and tissue specificity^14,15^. Aberrant expression of circRNAs has been observed in numerous diseases, such as cancers, cardiovascular diseases, and neurodegeneration, and plays active roles in the development and progression of diseases^16,17^. It was reported that circRNA ACVR2A is downregulated in BCa tissues and cells and that overexpression of circRNA ACVR2A suppresses BCa cell proliferation and metastasis by sponging miR-626 to regulate EYA4 expression^18^. CircRNA Cdr1as sensitizes BCa to cisplatin treatment through the miR-1270/APAF1 axis^19^. Notably, circRNA may be an important novel regulator in BCa. Nevertheless, a comprehensive understanding of the roles of circRNAs in BCa is lacking, and whether other circRNAs are involved in the disease remains largely unknown.

In this study, we identified the differentially expressed circRNAs in BCa compared to those in normal controls by circRNA microarray and then focused on a downregulated circRNA named circST6GALNAC6 for further functional exploration in BCa. Our study revealed a new molecular mechanism by which circST6GALNAC6, regulated by the transcription factor SP1, could suppress BCa metastasis by sponging miR-200a-3p to modulate the STMN1/EMT signalling axis, providing new ideas and potential targets for the development of therapeutic strategies for BCa.

## Materials and methods

### Human BCa specimen

Human BCa specimens were collected from 30 diagnosed BCa patients during surgery at The Second Xiangya Hospital of Central South University. The detailed information of the BCa patients was shown in Supplement table 1. Adjacent non-tumour tissues were obtained simultaneously from the same patients. Patients did not receive preoperative treatments. All patients were informed of the study and consented with a signed written form. The study was approved by the Ethics Committee of the Second Xiangya Hospital of Central South University. All specimens were immediately snap-frozen in liquid nitrogen and stored at −80 °C before further analysis.

### Cell culture and TGF-β1 treatment

Human bladder carcinoma cell lines (T24, J82, UM-UC-3, 5637 and SW780) and a normal human uroepithelial cell line (SV-HUC-1) were obtained from the Cell Bank of the Chinese Academy of Sciences (Shanghai, China). T24 and 5637 cells were grown in RPMI-1640 medium (Thermo Fisher Scientific, MA, USA), and other cells (J82, UM-UC-3, SW780, SV-HUC-1) were cultured in Dulbecco’s modified Eagle’s medium (DMEM) (Gibco, CA, USA). Both median were supplemented with 10% foetal bovine serum (Thermo Fisher Scientific, MA, USA) and 1% penicillin-streptomycin. All cells were maintained in a standard cell culture incubator at 37 °C with 95% humidity and 5% CO_2_. To induce EMT, J82 and UM-UC-3 cells were treated with TGF-β1 at a final concentration of 2.5 ng/ml for 24 h.

### Plasmids and transfection

Full-length SP1, STMN1, or circST6GALNAC6 was cloned into the lentivirus vector pLV-CMV. The constructs were transfected into HEK293T cells with the helper vectors pSPAX2 and pMD2G to generate lentivirus. The lentivirus was used to infect BCa cells for overexpression. hsa-miR-200a-3p mimics, inhibitors, SP1-sh1, SP1-sh4, and STMN1-shRNA were synthesized and purchased from Genema (Shanghai, China). Cell transfection was performed using Lipofectamine 3000 (Invitrogen, USA) according to the manufacturer’s protocol. Briefly, cells were grown to 60–80% confluence, and approximately 1 μg construct and 1 μl Lipofectamine 3000 were added to the media. Cells were harvested for further analysis 48 h after transfection. Stable cell lines were selected with appropriate antibiotics (puromycin, 3 μg/μl, Sigma) for one week after virus infection or construct transfection.

### Microarray analysis

TRIzol reagent (Invitrogen, Missouri, USA) was used to isolate total RNA from human tissues based on the manufacturer’s instructions. RNA integrity and contamination were determined by electrophoresis. rRNAs were removed, and circRNAs were enriched by using the rRNA Removal Kit and CircRNA Enrichment Kit (Illumina, CA, USA), respectively. The sequence libraries were constructed using the TruSeq Stranded Total RNA Library Prep Kit (Illumina, CA, USA) following the manufacturer’s guidance. The libraries were sequenced by a HiSeq 4000 Sequencer (Illumina, CA, USA). Qualified reads controlled by Q30 were aligned to human genome references by using TopHat2 software. CircRNAs were identified by the UROBORUS and CIRI tools. The relative expression levels of circRNAs were quantified according to the back-spliced reads per million mapped reads. The circRNAs with an absolute log2-fold change (FC) value >1 and a *P*-value < 0.05 were considered differentially expressed^20^.

### RNA extraction and qRT-PCR

TRIzol reagent (Invitrogen, Missouri, USA) was used to isolate total RNA from cultured cells according to the manufacturer’s instructions. DNase I was included in the lysis buffer to avoid DNA contamination. CircST6GALNAC6 and its linear counterpart ST6GALNAC6 mRNA were digested by the RNase R. 2 μg of total RNA was incubated for 30 min at 37 °C with 0.5 μL 10×RNase R Reaction Buffer and 0.2 μL RNase R or not. The relative expression levels of circST6GALNAC6 and ST6GALNAC6 mRNA were then measured by qRT-PCR.

Total RNA (1–2 μg) from each sample was used for reverse transcription and then amplified by PCR with standard kits (Invitrogen, Missouri, USA). Relative expression levels of circST6GALNAC6, miR-200a-3p, or SP1, STMN1 mRNA were normalized to U6 or GAPDH mRNA expression, respectively, as internal controls. The following primers were used:

circST6GALNAC6 forward primer (divergent): 5′- CATCATTCATGCGGATTGTACACT-3′;

circST6GALNAC6 reverse primer (divergent): 5′- TTTGACGACCTCTTCCGGGGTGAGA-3′;

circST6GALNAC6 forward primer (convergent): 5′- CCACGACGCGGTAGGTGG -3′;

circST6GALNAC6 reverse primer (convergent): 5′- GCCACCAGTGTGTGATTGTC-3′;

miR-200a-3p forward primer: 5′-TAACACTGTCTGGTAACGATGT-3′;

miR-200a-3p reverse primer: 5′-CATCTTACCGGACAGTGCTGGA-3′;

SP1 mRNA forward primer: 5′-TTG AAA AAG GAG TTG GTG GC-3′;

SP1 mRNA reverse primer: 5′-TGC TGG TTC TGT AAG TTG GG-3′;

STMN1 forward primer: 5′-CCAGGTGAAAGAACTGGAGA-3′;

STMN1 reverse primer: 5′-CTTCTGAATTTCCTCCAGGG-3′;

U6 forward primer: 5′-CTCGCTTCGGCAGCACA-3′;

U6 reverse primer: 5′-AACGCTTCACGAATTTGCGT-3′;

GAPDH forward primer: 5′-GAGTCAACGGATTTGGTCGTT-3′; and

GAPDH reverse primer: 5′-TTGATTTTGGAGGGATCTCG-3′.

### Western blotting

RIPA lysis buffer (ThermoFisher, MI, USA) was utilized to extract proteins from tissues or cells according to a standard protocol. The protein concentration of each sample was measured using the Pierce™ BCA Protein Assay Kit (Thermo Fisher, MI, USA). Equal amounts of protein were loaded into SDS-polyacrylamide gels and separated through electrophoresis. Later, the proteins were transferred from the gels to PVDF membranes (Sigma-Aldrich, USA). The membranes were blocked with 3% BSA for half an hour at room temperature and then incubated with primary antibodies overnight at 4 °C. On the next day, the membranes were washed with TBST 3 times before incubation with specific secondary antibodies for 1 h at room temperature. Signals were detected by using a standard ECL kit. The primary antibodies used in the study were as follows: anti-SP1 (1:2000; Cell Signalling, USA); anti-N-cadherin (1:1000; Abcam, USA); anti-vimentin (1:2000; Abcam, USA); anti-E-cadherin (1:1000; Abcam, USA); anti-STMN1 (1:1000; Abcam, USA); and anti-β-actin (1: 5000, Abcam, USA).

### Immunohistochemical (IHC) analysis

Tissues were fixed in 10% formalin at 4 °C overnight and embedded in paraffin. Then, paraffin sections (4-μm thick) were permeabilized with 0.5% Triton X-100 in PBS for 1 h at room temperature, followed by incubation with specific primary antibodies at 4 °C overnight (vimentin, E-cadherin, STMN1). The sections were washed with PBS 3 times and then stained with HRP-conjugated secondary antibodies for 2 h at room temperature. Slides were imaged with an LSM510 confocal microscope system (Zeiss). The following primary antibodies were used: anti-vimentin (1:500; Abcam, USA), anti-E-cadherin (1:400; Abcam, USA), and anti-STMN1 (1:400; Abcam, USA). The following secondary antibodies were used: HRP goat anti-mouse secondary antibody (1:500, Abcam, USA) and HRP goat anti-rabbit secondary antibody (1:500, Abcam, USA).

### RNA fluorescence in situ hybridization (FISH)

Cy5-labelled circST6GALNAC6 probes and Cy3-labelled miR-200a-3p probes were designed and synthesized by BersinBi (Guangzhou, China). The probe signals were determined with a FISH Kit (BersinBi, Guangzhou, China) according to the manufacturer’s guidelines. Briefly, paraffin sections (4-μm thick) and BCa cells were permeabilized with 0.5% Triton X-100 in PBS for 5 min at room temperature. Fluorescence-labelled specific probes for circST6GALNAC6 and miR-200a-3p were incubated at 37 °C overnight in a dark chamber. The next day, the samples were washed with PBS, and DAPI was added for nuclear staining. Images were acquired using a fluorescence microscope (Zeiss).

### Immunofluorescence (IF) staining

Cells were fixed in 4% paraformaldehyde (PFA) first for 10–15 min at room temperature and then permeabilized with 0.5% Triton X-100 in PBS for 5 min at room temperature. Cells were then blocked with 3% BSA for an hour at room temperature followed by incubation with specific primary antibodies overnight at 4 °C. The next day, the cells were washed with PBS and then incubated with fluorophore-conjugated secondary antibodies for 1 h at room temperature. Stained cells were imaged with an LSM510 confocal microscope system (Zeiss). The following primary antibody was used: anti-SP1 (1:400; Abcam, USA).

### MTT assay

Transfected cancer cells were plated in a 96-well plate at a density of 5000 cells/well and grown for different times, as indicated in the figures, followed by 3-(4,5-dimethylthiazol-2-yl)-2,5-diphenyltetrazolium bromide (MTT) incubation. Ten microlitres of MTT (5 mg/ml) was added and incubated for 3 h at 37 °C. Afterwards, 100 μl of detergent reagent was added to terminate the reaction. The absorbance at 570 nm in each condition was analysed.

### Colony formation assay

Transfected cancer cells were seeded in a 12-well culture plate and grown at 37 °C for 7 days. The colonies were fixed with 10% methanol for 10 min and stained with 1% crystal violet for 10 min followed by imaging with the EVOS FL Imaging System. The number of colonies was counted with ImageJ.

### Transwell assay

Transfected cancer cells were cultured in serum-free culture medium on the upper chamber of Transwell filter chamber filters that were pre-coated with Matrigel. Culture medium with 10% FBS was placed in the lower chamber as a chemoattractant. After 24 h of incubation at 37 °C, cells inside the upper chamber were discarded. Cells migrating to the lower chamber were fixed in 4% PFA, stained with 0.2% crystal violet and counted. For the invasion assay, the upper chamber was pre-coated with extracellular matrix (BD Biosciences, USA), a soluble basement membrane matrix, and cells invading the lower chamber after 24 h were fixed in 4% paraformaldehyde, stained with 0.2% crystal violet and counted.

### Chromatin immunoprecipitation (ChIP) assay

ChIP was performed using a commercial ChIP kit (Cell Signalling Technology, USA) according to the manufacturer’s protocol. For each chromatin immunoprecipitation assay, 5 μg of anti-SP1 or 1 μL of normal rabbit IgG was used as a negative control. After immunoprecipitation, chromosomal DNA was purified. The circST6GALNAC6 promoter region was detected by using PCR. The primers used for analysis were forward: 5′-ACTGACCTGCATCCTTCT-3′ and reverse: 5′- GGTGGCACATGCCTGTAA-3′.

### MS2-TRAP (MS2-tagged RNA affinity purification)

MS2-binding sequences (MS2bs) were infused with circST6GALNAC6. Cells were transfected with MS2-circST6GALNAC6 or MS2 control vector together with MS2bs-GFP using Lipofectamine 3000. After 2 days, transfected cells were lysed in lysis buffer (50 mM Tris-HCl, 150 mM NaCl, 2 mM EDTA, 1% NP-40, 0.5% sodium deoxycholate) containing RNase inhibitors and protease inhibitors (Thermo Scientific, Waltham, MA, USA). The extracted proteins were incubated with relevant antibodies (anti-GFP1 or IgG as a control) (Millipore, USA) overnight at 4 °C and then pulled down with protein a G Sepharose 4 Fast Flow suspension (Millipore, USA). The beads were digested with proteinase K (Sangon, Shanghai, China) for 1 h followed by RNA purification with TRIzol reagent (Invitrogen, Missouri, USA). Quantitative RT-PCR was performed to examine the RNA yield. The primers are listed in the qRT-PCR section.

### RNA Immunoprecipitation (RIP) assay

Transfected cells were lysed in lysis buffer (50 mM Tris-HCl, 150 mM NaCl, 2 mM EDTA, 1% NP-40, 0.5% sodium deoxycholate) containing RNase inhibitors and protease inhibitors (Thermo Scientific, Waltham, MA, USA). The extracted proteins were incubated with relevant antibodies (anti-AGO2, 1:1000 dilution, and IgG as a control) (Abcam, MA, USA) overnight at 4 °C and then pulled down with a protein G Sepharose 4 Fast Flow suspension (GE Amersham, Little Chalfont, UK). The beads were digested with proteinase K (Sangon, Shanghai, China) for 1 h followed by RNA purification with TRIzol reagent (Invitrogen, Missouri, USA). Quantitative RT-PCR was performed to examine the RNA yield. The primers are listed in the qRT-PCR section.

### Dual luciferase assay

The wild-type sequences or mutated binding sites of miR-200a-3p in the 3′ UTR of STMN1 and circST6GALNAC6 were cloned downstream of the luciferase reporter vector (psiCHECK2). Cells were first seeded in 24-well culture plates overnight, and then recombinant constructs were transfected into 293T cells using Lipofectamine 3000 together with miR-200a-3p mimics or mimics-NC. To analyse the interaction of SP1 and the circST6GALNAC6 promoter, the circST6GALNAC6 promoter, named pGL2000 (−2000~+1 nt) was cloned upstream of the luciferase reporter vector (pGL3-base) and transfected into 293T cells with the SP1 overexpression vector. To identify the core promoter region responsible for constitutive expression of the ST6GALNAC6 gene, serial truncated fragments of promoter from 3′-end were synthesised and subcloned into pGL3-base vector. Four successive truncated constructs from ST6GALNAC6 promoter named pGL1500 (−500 to −2000 nt), pGL1000 (−1000 to −2000 nt), pGL500 (−1500 to −2000 nt), pGL200 (−1800 to −2000 nt). Next, three mutation fragments of the Sp1 binding sites named pGL-Mut1 (−1238 to −1247 nt), pGL-Mut2(−1194 to −1208 nt), pGL-Mut3 (−469 to −478 nt) and pGL-Mut (−109 to −118 nt) were generated and transfected into 293T cells with the SP1 overexpression vector. After 48 h, the cells were harvested in Reporter Lysis Buffer from a commercial kit (Promega, WI, USA), and relative luciferase activities were measured.

### Nude mouse xenograft model

Five-week-old nude mice were purchased from Hunan SJA Laboratory Animal Co., Ltd. and kept under laboratory conditions. All animal experiments and protocols were reviewed and approved by the Animal Care and Use Committee of Central South University. Stable BCa cells (UM-UC3) expressing NC, circST6GALNAC6, or circST6GALNAC6 plus STMN1 shRNA or overexpression of circST6GALNAC6 and miR-200a-3p were injected into 5-week-old nude mice (2 × 10^6^ cells per mouse, five mice per group) through the tail vein. After 1.5 months, the lung tissues were excised under anaesthesia, and the numbers of macroscopically visible lung metastatic nodules were counted and validated by microscopy assessment of haematoxylin and eosin (H&E)-stained sections.

### Statistical analysis

All experiments were performed with at least three biological replicates. All statistical analyses were performed in GraphPad Prism 7. Statistical significance was determined by unpaired Student’s *t* test (two groups) or one-way ANOVA (multiple groups). The data are presented as the mean ± SD.

## Results

### CircST6GALNAC6 was downregulated in BCa tissues and cells

**Fig. 1:**
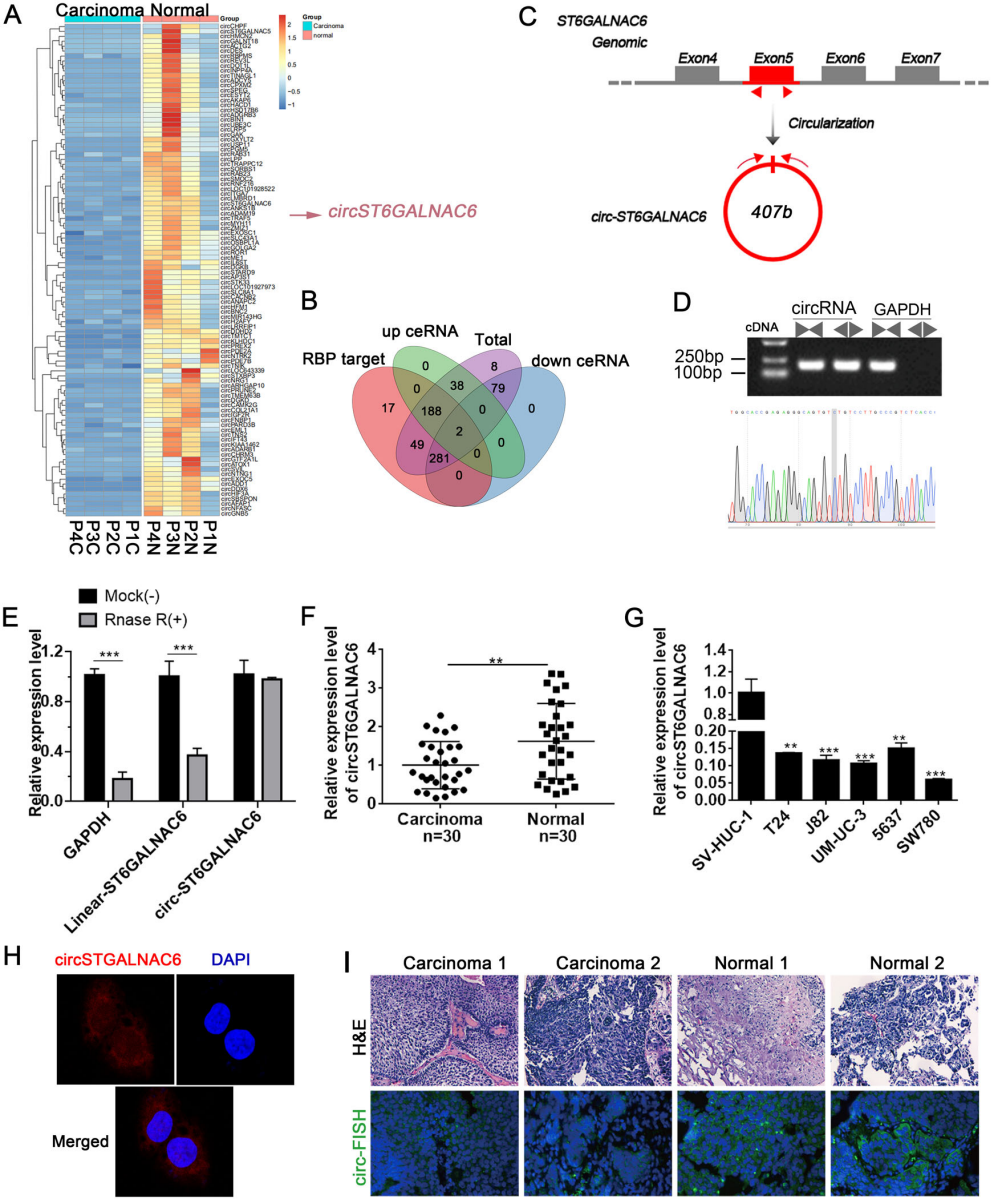
CircST6GALNAC6 is downregulated in bladder cancer tissues and cells. **A** Heatmap exhibiting the top 100 downregulated circRNAs, which are displayed on a scale from blue (small change) to orange (large change), between four bladder cancer (BCa) tissues and adjacent normal tissues from 4 human patients. **B** Schematic illustration shows the number of circRNAs that bind with RBPs or miRNAs. **C** Schematic representation of circST6GALNAC6 formation. **D** Gel electrophoresis shows that circST6GALNAC6 can be amplified by divergent primers using total cDNA. Sanger sequencing validates its splicing junction. GAPDH served as a control. **E** CircST6GALNAC6 resistance to Rnase R was determined by qRT-PCR assay. GAPDH served as an endogenous control. **F**, **G** circST6GALNAC6 expression was analysed by qRT-PCR in 30 BCa tissues and 5 BCa cells (T24, J82, UM-UC-3, 5637 and SW780) and compared with 30 adjacent normal tissues and a normal human uroepithelial cell line (SV-HUC-1), respectively. **H**, **I** Representative images of H&E staining (**H**) and FISH staining (**I**) to analyse circST6GALNAC6 levels in two BCa tissues (Carcinoma 1/2) and normal tissues. **G** FISH assay was conducted to detect the expression distribution of circST6GALNAC6 in UM-UC-3 cells. ***P < 0.001 and **P < 0.01.

To investigate the circRNA expression profile in four pairs of BCa samples (BCa and matched adjacent non-tumour tissues), we used the SBC Human circRNA microarray. A total of 881 dysregulated circRNAs were identified in BCa tissues, of which 300 circRNAs were significantly upregulated and 581 circRNAs were significantly downregulated (data not shown). The clustered heatmap in Fig. 1A shows the top 100 downregulated circRNAs (Fig. 1A). Furthermore, the potential targets of 363 downregulated circRNAs were analysed, revealing that 283 circRNAs may exert functions by modulating RNA-binding proteins (RBPs) or sponging miRNAs, while 79 may exert functions only by sponging miRNAs (Fig. 1B). In addition, we selected 3 downregulated circRNAs to verify the microarray results. The results showed that the expression of these 3 circRNAs was consistent with the microarray results obtained by qRT-PCR analysis in BCa tissues and cells (Supplement Fig. 1). Among them, we found that circST6GALNAC6 was derived from back-splicing of exon 5 in the ST6GALNAC6 (NM_013443) gene, with a length of 407 nt (Fig. 1C), which is consistent with the circbase database annotation of hsa_circ_0088708 (http://www.circbase.org/). We detected the expression of this transcript as circST6GALNAC6 by amplifying it from the cDNA of J82 cells using the divergent primer strategy and the splice junction of circST6GALNAC6 was verified by Sanger sequencing (Fig. 1D), Because circRNAs are resistant to RNase R treatment, we performed RNase R digestion assay. Both linear RNA transcripts (ST6GALNAC6 and GAPDH mRNA) were significantly degraded compared with circST6GALNAC6, further validating the circular nature of the circST6GALNAC6 transcript (Fig. 1E). Notably, circST6GALNAC6 was significantly decreased in another 30 paired BCa samples and 5 BCa cells (compared to SV-HUC-1 cells) (Fig. 1F, G) by qRT-PCR analysis. Furthermore, H&E and FISH analysis showed that BCa tissues have lower levels of circST6GALNAC6 than normal tissues (Fig. 1H, I). Notably, circST6GALNAC6 was expressed in the cytoplasm and nucleus of UM-UC-3 cell, but mainly expressed in the cytoplasm. Together, these results suggest that circST6GALNAC6 downregulation is common in BCa tissues and cells.

### Overexpression of circST6GALNAC6 inhibited the proliferation and migration of bladder cancer cells

**Fig. 2:**
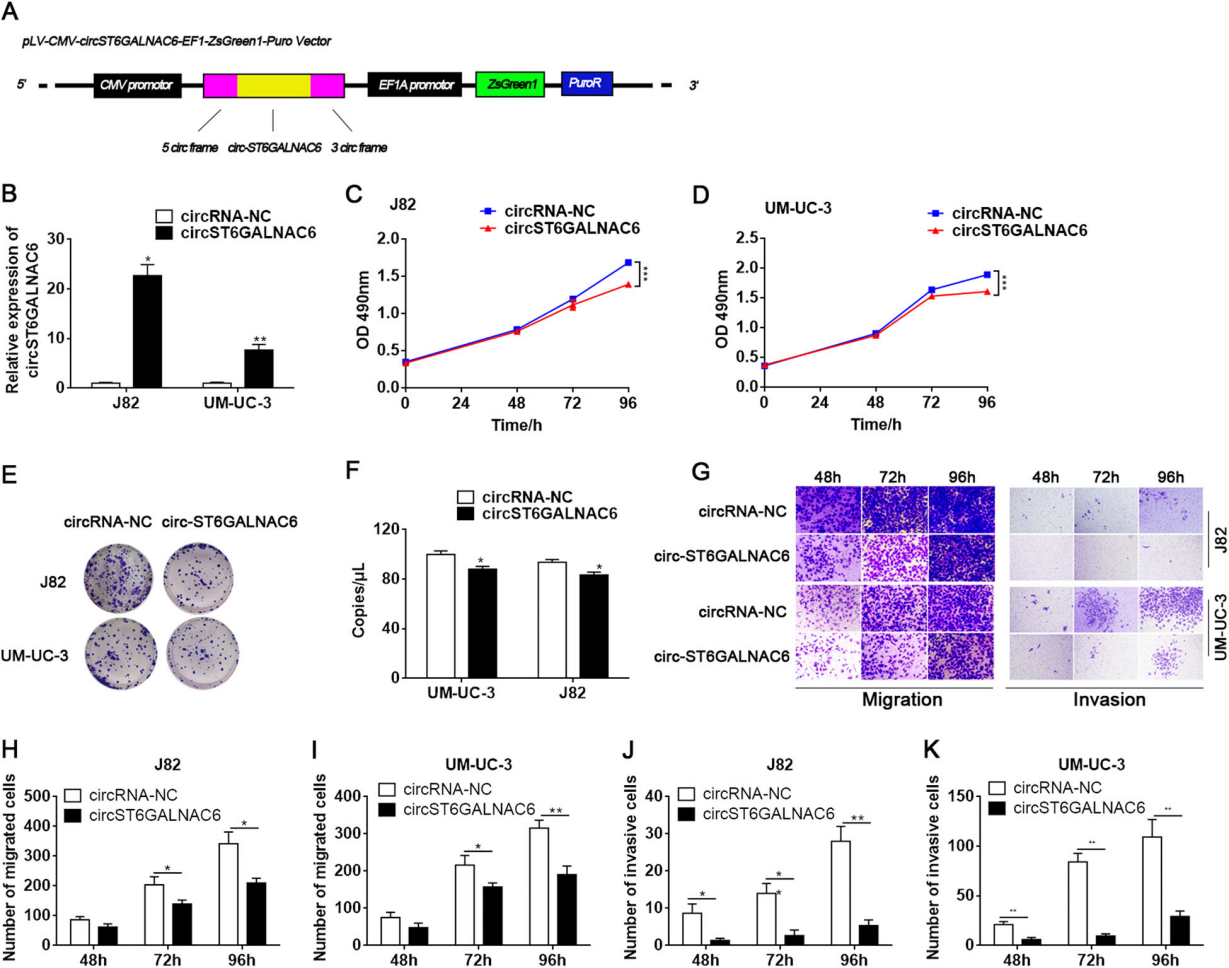
Overexpression of circST6GALNAC6 inhibited the proliferation, migration and invasion of bladder cancer cells. **A** Schematic representation of the plasmid overexpressing circST6GALNAC6. **B** Relative circST6GALNAC6 levels in cells transfected with circRNA-NC or circST6GALNAC6 were detected by qRT-PCR. **C**, **D** MTT assay to measure the cell viability of cancer cells transfected with circRNA-NC or circST6GALNAC6. **E**, **F** Cell proliferation abilities were detected by colony formation assays in transfected J82 and UM-UC-3 cells. **G**–**K** Cell migration and invasion abilities were measured with Transwell assays. ***P < 0.001, **P < 0.01 and *P < 0.05.

The roles of circST6GALNAC6 in BCa were investigated. First, we constructed circST6GALNAC6-overexpressing lentiviruses with circular frames and circST6GALNAC6 sequences (Fig. 2A) and found that circST6GALNAC6 was markedly overexpressed in J82 and UM-UC-3 cells (Fig. 2B). With the MTT assay, we showed that overexpression of circST6GALNAC6 suppressed J82 and UM-UC-3 cell proliferation (Fig. 2C, D). Consistently, colony formation assays demonstrated that overexpression of circST6GALNAC6 significantly decreased the number of colonies formed in J82 and UM-UC-3 cell lines (Fig. 2E, F). In addition, we measured the migration and invasion abilities of cancer cells with a Transwell assay. As shown in Fig. 2G-K, overexpression of circST6GALNAC6 drastically inhibited cell migration and invasion compared with circRNA-NC. These data indicated that overexpression of circST6GALNAC6 suppresses the proliferation, migration and invasion of BCa cells in vitro.

### SP1 bound the circST6GALNAC6 promoter and activated its transcription

**Fig. 3:**
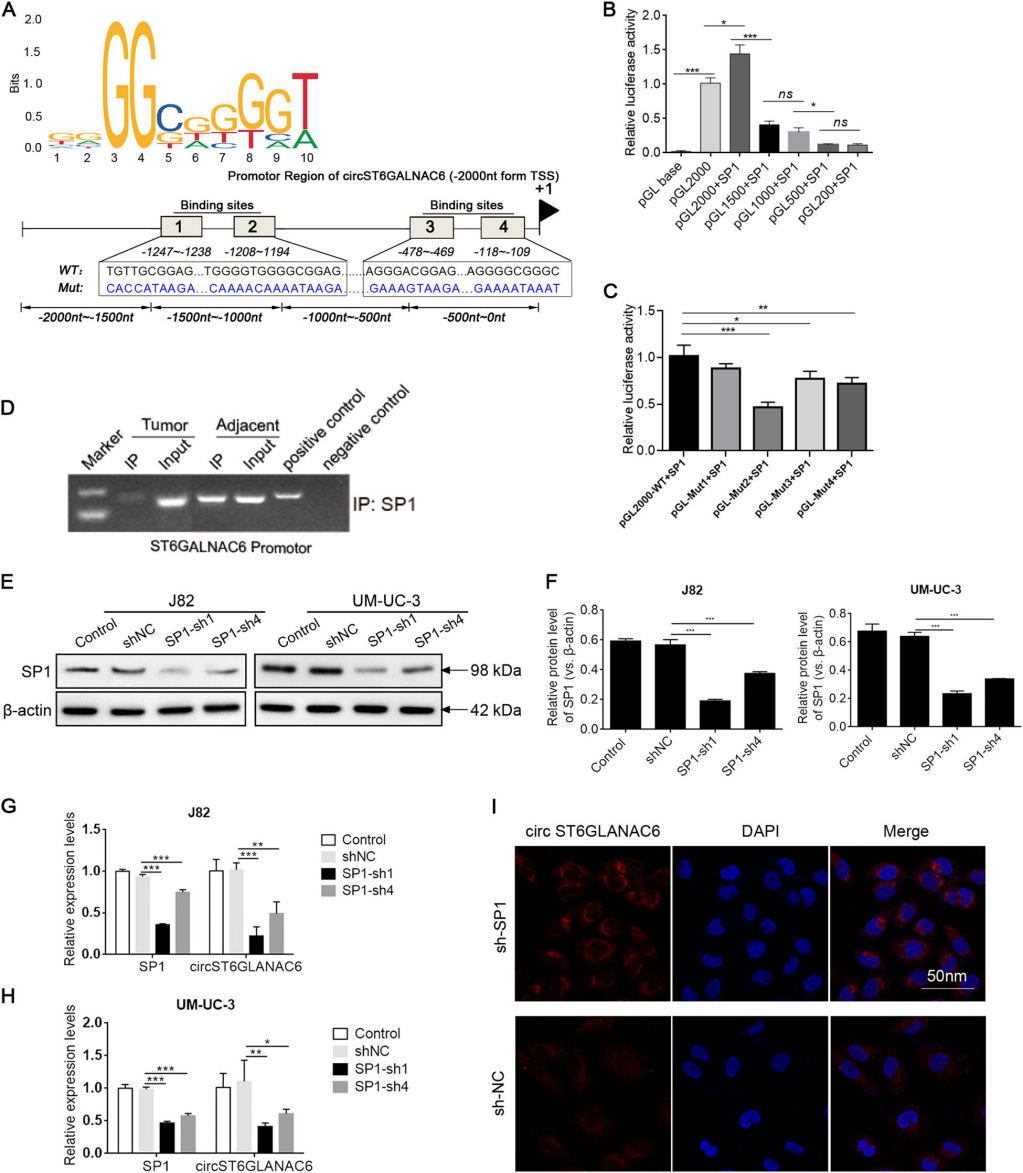
SP1 bound to the circST6GALNAC6 promoter and activated its transcription. **A** Schematic representation of the binding region of SP1 specific to the promoter of circST6GALNAC6. **B** A luciferase reporter assay was performed to detect the activity of circST6GALNAC6 in 293T cells co-transfected with SP1. The firefly luciferase activity of each sample has been normalized by the Renilla luciferase activity and has been presented as percentage activity of the pGL-base containing SV40 promoter. **C** The dual luciferase reporter assay was used to detect the transcription activity of pGL2000WT + SP1, pGL-Mut1 + SP1, pGL-Mut2 + SP1, pGL-Mut3 + SP1, pGL-Mut4 + SP1. The firefly luciferase activity of each sample has been presented as percentage activity of the pGL2000WT + SP1. **D** ChIP-PCR was used to analyse the binding of SP1 with the circST6GALNAC6 promoter in bladder cancer tissues and normal tissues. **E**, **F** SP1 protein levels were detected by western blotting in cells transfected with NC or SP1 shRNA. **G**, **H** Relative SP1 and circST6GALNAC6 mRNA levels in cells transfected with NC or SP1 shRNA were measured using qRT-PCR. **I** circST6GALNAC6 expression was measured by FISH in cells transfected with NC or SP1 shRNA. ***P < 0.001, **P < 0.01 and *P < 0.05.

We then explored the mechanisms underlying the abnormal expression of circST6GALNAC6 in BCa. Bioinformatics prediction using the JASPAR database (http://jaspar.genereg.net/) shows that there are twelve binding sites within SP1(when the Relative score ≥0.85), a transcription factor, and the circST6GALNAC6 promoter region (Fig. 3A). The luciferase activity of PGL2000 was significantly induced by SP1 (Fig. 3B). However, the truncation from 3′-end led to a gradual decrease of the luciferase activity from pGL2000 to pGL200. In addition, there is a significant decrease of the luciferase activity in pGL1500 + SP1 and pGL500 + SP1 group, which suggested the core promoter of ST6GALNAC6 gene was located at the region from −1 to −500 nt and −1000 to −1500 (Fig. 3B). Moreover, four Sp1 binding regions in the core promote were mutated, as shown in Fig. 3A. The dual luciferase reporter assays results suggested that the luciferase activity of pGL-Mut1 + SP1, pGL-Mut2 + SP1, pGL-Mut3 + SP1, pGL-Mut4 + SP1 reduced approximately 12%, 53%, 23%, 28%, respectively, compared with pGL2000 + SP1 (Fig. 3C). Consistently, ChIP demonstrated that the SP1 antibody was effectively precipitated with the region of the circST6GALNAC6 promoter in BCa tissues (Fig. 3D). Furthermore, we transfected cells with short interfering RNAs (SP1-sh1 and SP1-sh4) that specifically targeted SP1. Western blotting assays showed that SP1 shRNAs successfully knocked down SP1 protein expression in J82 and UM-UC-3 cells (3E-G). Moreover, SP1 knockdown significantly downregulated the expression of circST6GALNAC6 by qRT-PCR and FISH analysis (Fig. 3H-J). Taken together, these results demonstrate that SP1 binds the circST6GALNAC6 promoter, especially fragment −1194 to −1208 nt and promotes its transcription.

### circST6GALNAC6 acted as a miR-200a-3p sponge in BCa cells

**Fig. 4:**
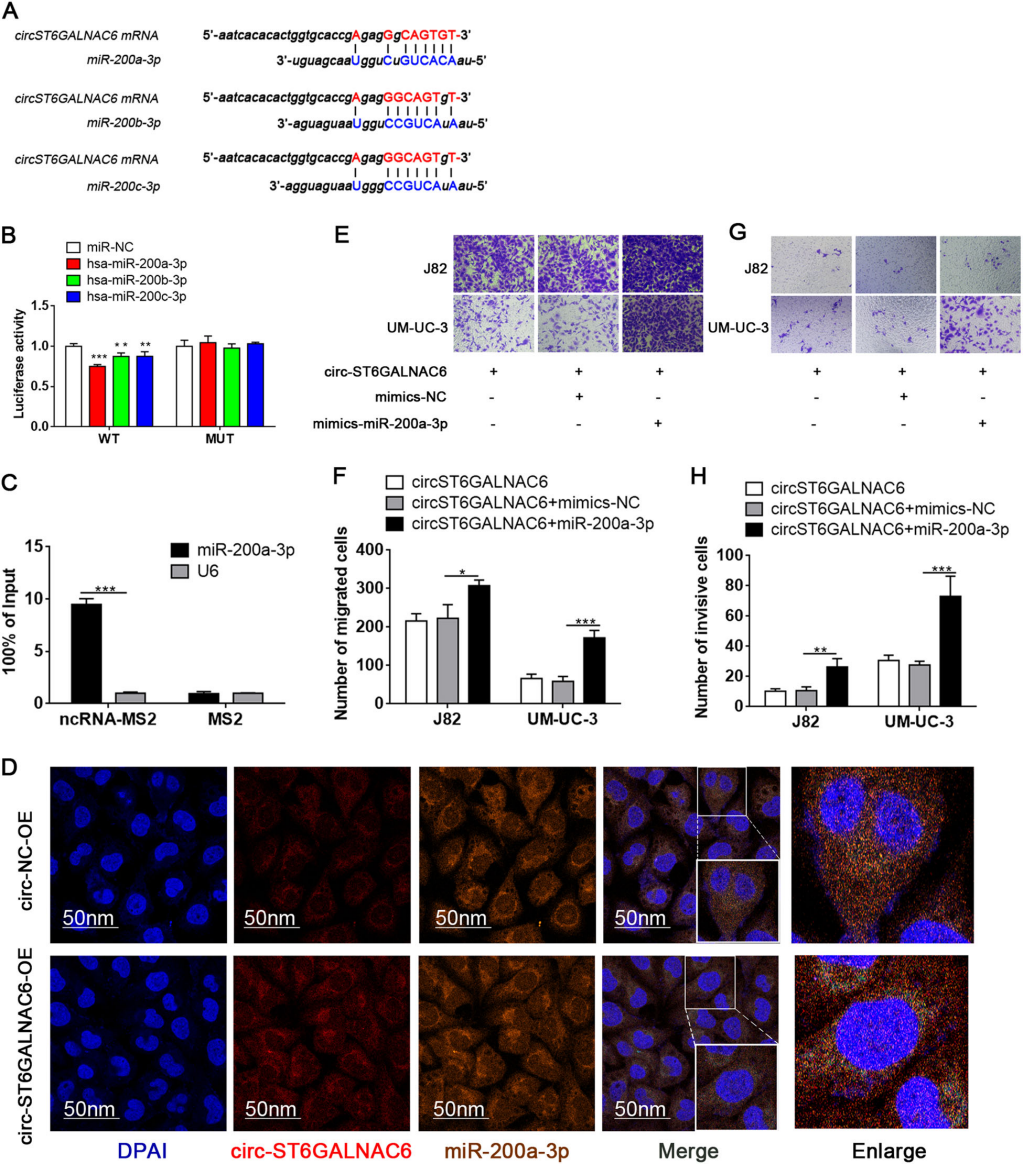
circST6GALNAC6 acted as a miR-200a-3p sponge. **A** The putative binding sites of miR-200a/b/c-3p on circST6GALNAC6 are shown. **B** A luciferase reporter assay was performed to detect the activity of circST6GALNAC6 in 293T cells co-transfected with miR-200a/b/c-3p or scramble (miR-NC) and wild-type (WT) circST6GALNAC6 or mutated (MUT) circST6GALNAC6. **C** MS2-TRAP to analyse the binding of circST6GALNAC6 with miRNAs. **D** Colocalization of circST6GALNAC6 and miR-200-3p was measured using FISH in transfected J82 and UM-UC-3 cells. **E**–**H** The migration and invasion of transfected cells were analysed by Transwell assay. ***P < 0.001, **P < 0.01 and *P < 0.05.

It has been shown that circRNAs exert functions by modulating RBPs or sponging miRNAs. Through bioinformatic analysis, we identified three members of the miR-200-3p family (miR-200a-3p, miR-200b-3p, miR-200c-3p) that could potentially bind with circST6GALNAC6 (Fig. 4A). To directly examine whether these miRNAs target circST6GALNAC6, we used a dual luciferase reporter assay. As shown in Fig. 4B, the luciferase activity of wild-type (WT) circST6GALNAC6 was decreased by the miR-200a/b/c-3p mimic. However, the luciferase activity of MUT circST6GALNAC6 was not affected by these three miRNA mimics (*P* > 0.05) (Fig. 4B). Furthermore, we found that MS2-circST6GALNAC6 only pulled down miR-200a-3p, but not miR-200b-3p and miR-200c-3p, by MS2-TRAP assay, suggesting that circST6GALNAC6 serves as a binding platform for miR-200a-3p in BCa cells (Fig. 4C). Furtherly, FISH experiments showed that circST6GALNAC6 and miR-200a-3p were preferentially co-localized in the cytoplasm, supporting the direct interaction of circST6GALNAC6 with miR-200a-3p (Fig. 4D).

To address whether circST6GALNAC6 inhibits the migration and invasion of BCa cells via sponging by miR-200a-3p, miR-200a-3p mimics were used to examine whether the effect of circST6GALNAC6 depletion can be rescued by miR-200a-3p mimics. The results showed that miR-200a-3p mimics substantially restored circST6GALNAC6 overexpression-mediated suppression of BCa cell migration and invasion abilities (Fig. 4E–H). Altogether, these data indicate that circST6GALNAC6 regulates the migration and invasion of BCa cells by sponging miR-200a-3p.

### miR-200a-3p promoted EMT by targeting STMN1

**Fig. 5:**
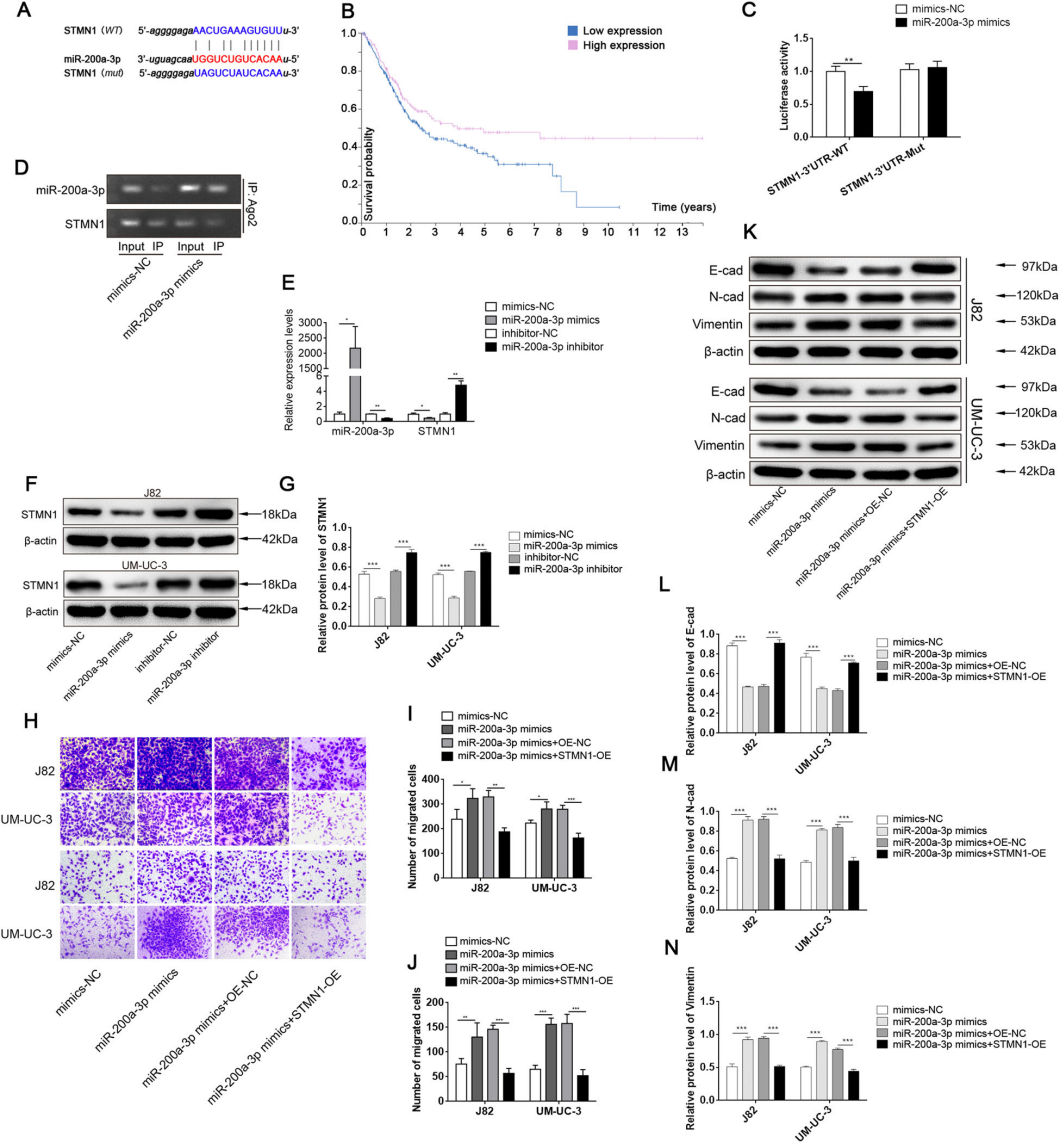
miR-200a-3p promoted EMT by targeting STMN1. **A** Complementary binding sites between miR-200a-3p and STMN1. **B** The survival curves for 406 bladder cancer patients are presented. **C** Relative luciferase activities of WT-STMN1 or MUT-STMN1 in miR-200a-3p transfected cells. **D** The RIP assay was performed to verify miR-200a-3p binding with STMN1 mRNA. **E** Relative miR-200a-3p and STMN1 mRNA levels in transfected cells were examined by qRT-PCR. **F**–**G** Relative STMN1 protein levels in transfected cells were detected by western blotting. **H**–**J** Transwell assay to analyse the migration and invasion of transfected cancer cells. **K**–**N** Relative protein levels of EMT-related proteins in transfected cells. ***P < 0.001, **P < 0.01 and *P < 0.05.

It is well known that miRNAs function by inhibiting the expression of downstream targets. By bioinformatic analysis (Starbase), we identified complementary binding sites between miR-200a-3p and STMN1 (Fig. 5A). We performed Kaplan–Meier survival analysis and found that STMN1 was positively associated with favourable survival in patients with BCa (Fig. 5B). A subsequent luciferase reporter assay revealed decreased luciferase intensity after co-transfection of miR-200a-3p mimics and a wild-type luciferase reporter (STMN1-3′ UTR-WT), while the mutated luciferase reporter (STMN1-3’ UTR-Mut) exerted no such effect (Fig. 5C). By RIP for AGO2, we observed endogenous miR-200a-3p and STMN1 mRNA pull-down in J82 cells, and STMN1 pull-down by AGO2 was reduced when miR-200a-3p was overexpressed (Fig. 5D). Moreover, we found that overexpression of miR-200a-3p greatly decreased STMN1 mRNA and protein levels, while miR-200a-3p inhibitor increased those levels (Fig. 5E–G). These results suggest that STMN1 may serve as a downstream factor of miR-200a-3p.

We further studied the function of the interaction between miR-200a-3p and STMN1 in BCa. Transwell assays revealed that miR-200a-3p markedly promoted the migration and invasion of BCa cells compared with the effects of miR-NC. However, this effect was abolished by co-overexpression of STMN1 (Fig. 5H–J). In addition, we measured the levels of EMT-related proteins by western blotting. The results indicated that miR-200a-3p mimics led to decreased expression of E-cadherin but increased expression of N-cadherin and vimentin in J82 and UM-UC-3 cell lines, indicating that the epithelial cells acquired mesenchymal properties. Nevertheless, these mesenchymal properties were reversed by co-overexpression of STMN1 (Fig. 5K–N).

### circST6GALNAC6 regulated EMT via miR-200a-3p/STMN1 signalling

**Fig. 6:**
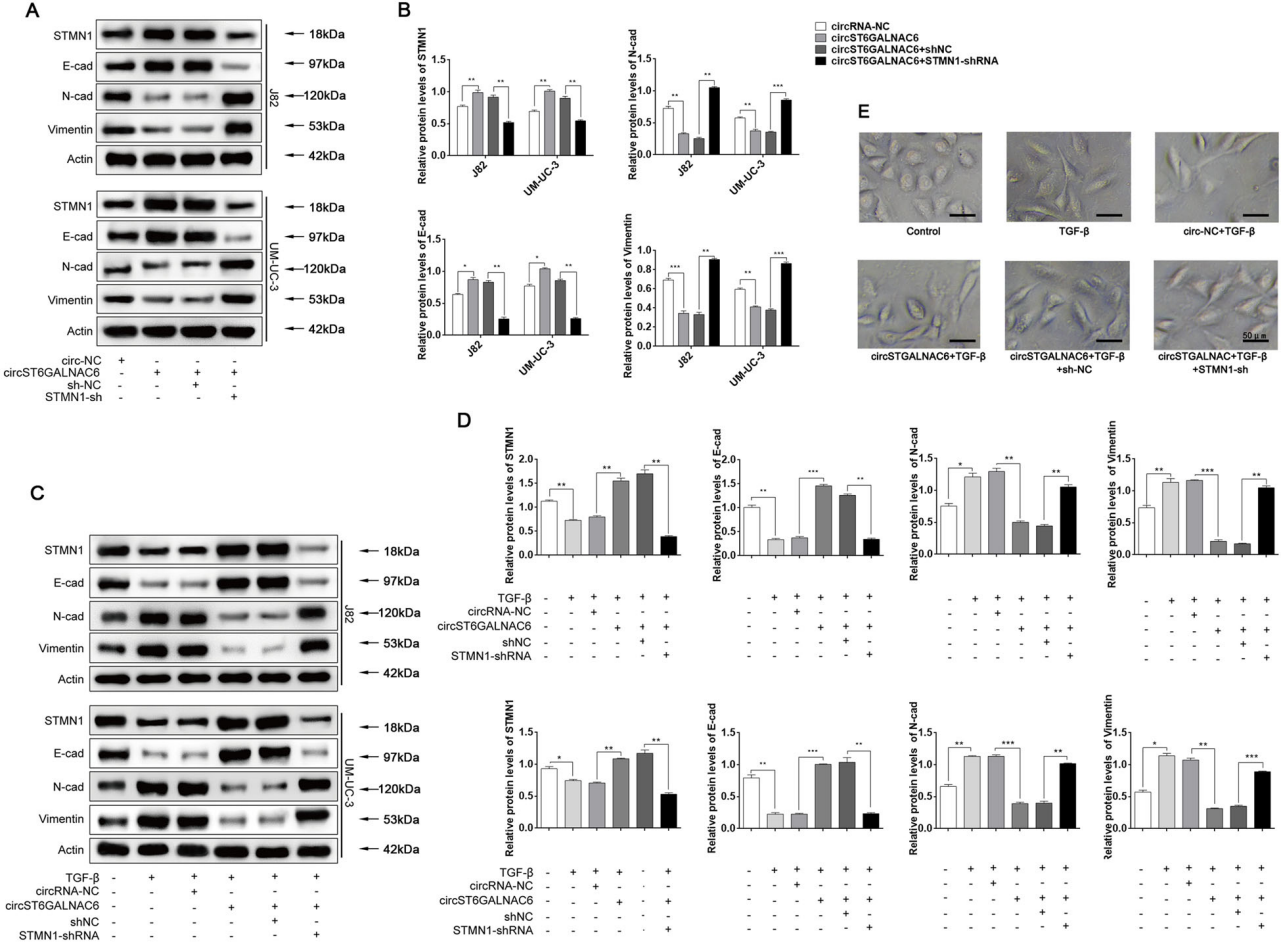
circST6GALNAC6 regulated EMT via miR-200a-3p/STMN1 signalling. **A**, **B** Relative protein levels of STMN1 and EMT-related proteins in transfected cells were detected by western blotting. **C**, **D** Relative protein levels of STMN1 and EMT-related proteins in transfected cells following TGF-β1 treatment. **E** UM-UC-3 cells were transfected with circST6GALNAC6 or circRNA-NC or circST6GALNAC6 plus STMN1-shRNA or shNC, and then treated with 50 ng/ml TGF-β1 for 72 day. And then, the cell morphology of UM-UC-3 was observed. ***P < 0.001, **P < 0.01 and *P < 0.05.

Our aforementioned results showed that circST6GALNAC6 sponges miR-200a-3p, while miR-200a-3p targets STMN1. To test whether circST6GALNAC6 regulated EMT through the miR-200a-3p/STMN1 axis, we manipulated their corresponding levels and examined the effects. Western blotting results showed that overexpression of circST6GALNAC6 increased STMN1 and E-cadherin protein levels. In contrast, circST6GALNAC6 decreased the levels of N-cadherin and vimentin (Fig. 6A, B), suggesting that circST6GALNAC6 suppresses EMT by activating STMNI. However, in cells co-transfected with circST6GALNAC6 and STMN1-shRNA, E-cadherin was significantly downregulated, while N-cadherin and vimentin were upregulated in these cells compared to cells transfected with circST6GALNAC6 and circRNA-NC (Fig. 6A, B), indicating that knockdown of STMN1 reverses the effects of circST6GALNAC6 on EMT. TGF-β1 has been proven to induce EMT and contribute to metastasis in various cells^21^. As expected, western blotting results showed that TGF-β1 significantly inhibited STMN1 and E-cadherin expression while promoting N-cadherin and vimentin expression (Fig. 6C, D). However, overexpression of circST6GALNAC6 decreased the levels of N-cadherin and vimentin while promoting STMN1 and E-cadherin expression, and knockdown of STMN1 reverses the effects of circST6GALNAC6 on EMT (Fig. 6C, D). Notably, overexpression of circST6GALNAC6 blocked TGF-β1-induced EMT, but this blockade was reversed by STMN1 shRNA (Fig. 6E). Together, these results clearly demonstrate that circST6GALNAC6 promotes EMT by increasing STMN1 levels, mostly by sponging miR-200a-3p.

### circST6GALNAC6 inhibited BCa metastasis in vivo via miR-200a-3p/STMN1

**Fig. 7:**
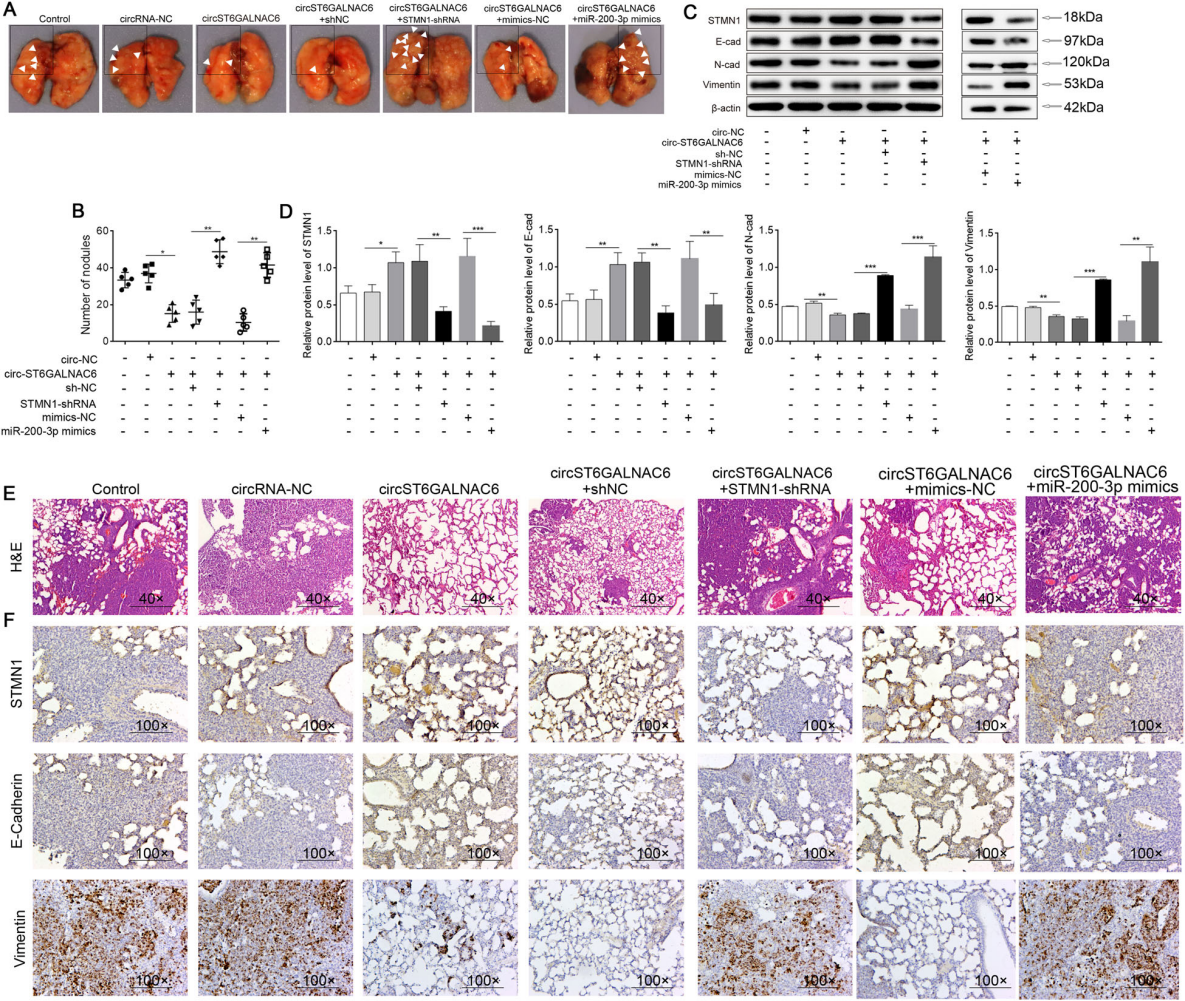
circST6GALNAC6 inhibited bladder cancer growth in vivo via miR-200a-3p/STMN1. **A** Representative images of lung metastatic nodules are shown. **B** The number of metastatic nodules was quantified in each group of mice. **C**, **D** STMN1 and EMT-related protein expression in tumours from each group of mice were detected by western blotting (**C**) and quantified (**D**). **E** H&E staining analysis of lung tissues from each group of mice. **F** STMN1, E-cadherin and vimentin expression was detected via IHC in each group of mice. ***P < 0.001, **P < 0.01 and *P < 0.05.

Finally, we evaluated the function of circST6GALNAC6 in BCa in vivo in established mouse xenograft models. We generated two stable UM-UC-3 cell lines overexpressing circST6GALNAC6 or circST6GALNAC6 plus STMN1-shRNA or overexpression circST6GALNAC6 plus miR-200a-3p and then injected them into nude mice through the tail vein. As shown in Fig. 7A, B, E, circST6GALNAC6 significantly decreased lung metastasis, which was reversed by treatment with silence STMN1 and overexpression of miR-200a-3p. In addition, the overexpression of circST6GALNAC6 upregulated STMN1 and E-cadherin but downregulated N-cadherin and vimentin expression, while knockdown of STMN1 or overexpression miR-200a-3p reversed these changes as shown by western blotting and IHC assays (Fig. 7D–F). Collectively, our results prove that circST6GALNAC6 suppresses EMT and BCa metastasis in vivo partly through the miR-200a-3p/STMN1 axis.

## Discussion

BCa is a common and lethal malignancy^1,2^. The pathogenesis and clinical manifestations are highly heterogeneous; thus, the treatment options are limited, and outcomes remain unsatisfactory^22,23^. CircRNAs are a class of circular non-coding RNAs that play critical roles in tumorigenesis^13^. Emerging evidence shows aberrant expression of circRNAs in a variety of cancers, including BCa^24–26^. Previous studies have implicated several circRNAs in BCa, such as circRNA BCRC-3, circ_0091017 and circPICALM^27–29^. However, as the BCa cohort was small, future studies with larger cohorts are necessary. In this study, through high-throughput RNA-seq, we provided a systematic and comprehensive dataset showing how circRNAs are involved in BCa. We identified 300 upregulated circRNAs and 581 downregulated circRNAs in human BCa tissues compared to non-tumour tissues. Moreover, we found a novel circRNA, circST6GALNAC6, that is derived from exon 5 of the ST6GALNAC6 gene. There are no previous reports about the role of circST6GALNAC6 in BCa. Here, we demonstrated for the first time that circST6GALNAC6 is greatly downregulated in BCa tissues and cells compared to expression in normal controls and that it plays an important role in BCa metastasis. Together with previous studies^24–26^, our work suggests that circRNAs can serve as diagnostic markers and therapeutic targets for certain diseases. However, there are limitations to our present study. First, only 4 paired BCa tissues were subjected to microarray analysis. Second, only 30 BCa tumour samples were analysed by qRT-PCR for confirmation. In future studies, we need to assess more BCa patient tissues to confirm the suppressor role of circST6GALNAC6, and it is also necessary to explore the function of other differentially expressed circRNAs in BCa.

MiRNAs have been widely implicated in diverse cellular processes and diseases^8,30^. The miR-200 superfamily is a key family of miRNAs that is largely involved in EMT and regulates cancer metastasis^31^. In this study, we found complementary binding sites between circST6GALNAC6 and miR-200a/b/c-3p. However, in BCa cells, we found that only circST6GALNAC6 bound with miR-200a-3p but not the other two members. Notably, we observed that miR-200a-3p promoted EMT and cancer cell migration in BCa and that circST6GALNAC6 exerted its functions by sponging miR-200a-3p. These results suggest that the miR-200 family has distinct functions in different cancers. It will be interesting to examine the role of circST6GALNAC6/miR-200 binding in other types of cancers. In addition, we showed that SP-1 is the upstream regulator of circST6GALNAC6 and activates the transcription of circST6GALNAC6 by binding its promoter. Of course, further evidence is required to conclude that circST6GALNAC6 is activated by other transcription factors.

It is well acknowledged that EMT is crucial for cancer progression, particularly metastasis^32^. Many signals and molecules are involved in this process, and STMN1 is one of them^33^. STMN1 is a phosphoprotein that functions to destabilize microtubules by facilitating microtubule depolymerization. STMN1 has been reported to be upregulated across many kinds of cancers, and its high expression correlates with a poor prognosis^34,35^. Previous studies have indicated that STMN1 inhibits EMT and the metastatic behaviours of epithelial cells^36^. In BCa, STMN1 was significantly decreased in TGFβ1-induced BCa cells and regulated TGFβ1-induced EMT, indicating that it serves as a target of miR-221^37^. Here, we showed similar functions of STMN1. Knockdown of STMN1 facilitated the migration, invasion and EMT of BCa cells in vitro and in vivo. Notably, we showed that miR-200a-3p directly targeted STMN1 and that miR-200a-3p regulated EMT through STMN1. Furthermore, silencing STMN1 reversed the effects of circST6GALNAC6 on EMT and tumour metastasis. Our study, together with previous work^38,39^, indicates that STMN1 is involved in complex regulatory networks and confers cell-type-specific regulation of cell function in different cancers.

In summary, we revealed that circST6GALNAC6 is downregulated in BCa and that it can efficiently sponge miR-200a-3p to inhibit STMN1. We also demonstrated that circST6GALNAC6 effectively suppresses BCa metastasis by regulating the miR-200a-3p/STMN1/EMT axis. These results shed light on the mechanisms of BCa and provide avenues for the development of diagnostic or therapeutic strategies for BCa.

## Supplementary information

Supplementary Table S1

Supplementary Figure Legends

supplementary fig1
