# Supplementary material for: circST6GALNAC6 suppresses bladder cancer metastasis by sponging miR-200a-3p to modulate the STMN1/EMT axis
Source: Cell Death Dis. 2021 Feb 10;12(2):168. doi: 10.1038/s41419-021-03459-4 (PMC7876104; doi:10.1038/s41419-021-03459-4)
Supplement: Supplementary file 1 — Supplementary Table S1 [file 41419_2021_3459_MOESM1_ESM.docx]

Supplementary Table S1. Clinical characteristics of 30 patients.

| Characteristics | N = 30 |
| --- | --- |
| Age at diagnosis |  |
| Median (range) | 63 (39-79) |
| Gender |  |
| Female | 6 (20%) |
| Male | 24 (80%) |
| Surgical resection |  |
| Radical cystectomy | 17 (56.7%) |
| Transurethral resection of bladder tumor | 13 (43.3%) |
| Primary/Recurrent |  |
| Primary | 26 (86.7%) |
| Recurrent | 4 (13.3%) |
| Degree of differentiation |  |
| Low grade | 9 (30%) |
| High grade | 21 (70%) |
| Tumor stage |  |
| T1N0M0 | 4 (13.3%) |
| T1aN0M0 | 3 (10%) |
| T2aN0M0 | 6 (20%) |
| T2aN1M0 | 6 (20%) |
| T2bN0M0 | 2 (6.7%) |
| T3aN0M0 | 4 (13.3%) |
| T3aN1M0 | 2 (6.7%) |
| T3aN2M0 | 2 (6.7%) |
| T4aN2M0 | 1 (3.3%) |
