# Supplementary material for: circST6GALNAC6 suppresses bladder cancer metastasis by sponging miR-200a-3p to modulate the STMN1/EMT axis
Source: Cell Death Dis. 2021 Feb 10;12(2):168. doi: 10.1038/s41419-021-03459-4 (PMC7876104; doi:10.1038/s41419-021-03459-4)
Supplement: Supplementary file 2 — Supplementary Figure Legends [file 41419_2021_3459_MOESM2_ESM.docx]

Supplement Figure 1. **circMYH11 and circPRUNE2 were upregulated in BCa tissues and cells.**

(A and B) The expression of circMYH11 and circPRUNE2 were analysed by qRT-PCR in 30 BCa tissues compared with 30 adjacent normal tissues. (C and D）The expression of circMYH11 and circPRUNE2 were analysed by qRT-PCR I n5 BCa cells (T24, J82, UM-UC-3, 5637 and SW780) compared with a normal human uroepithelial cell line (SV-HUC-1). ***P < 0.001, **P < 0.01 and *P < 0.05.
