# Supplementary figures and images for: circST6GALNAC6 suppresses bladder cancer metastasis by sponging miR-200a-3p to modulate the STMN1/EMT axis
Source: Cell Death Dis. 2021 Feb 10;12(2):168. doi: 10.1038/s41419-021-03459-4 (PMC7876104; doi:10.1038/s41419-021-03459-4)

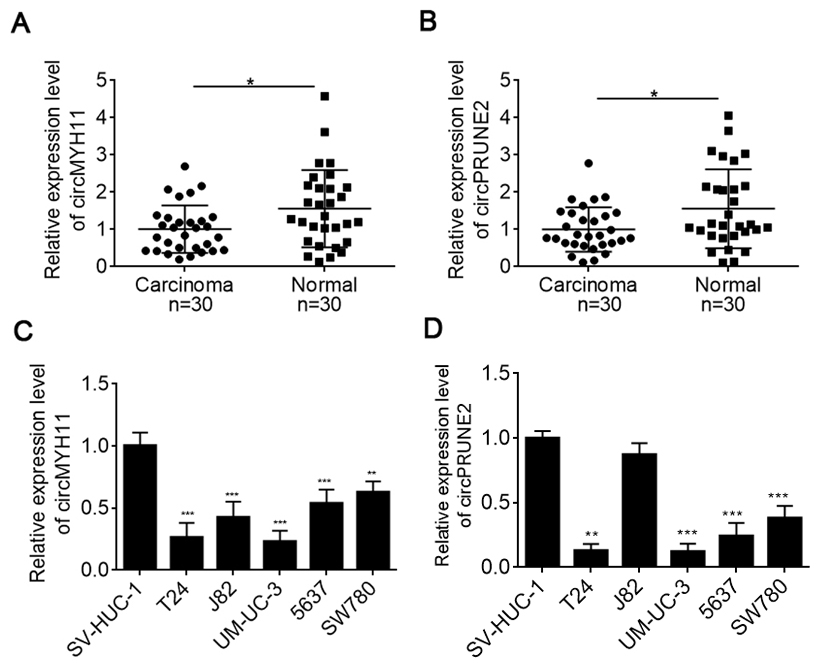

Supplement: Supplementary file 3 — supplementary fig1 [file 41419_2021_3459_MOESM3_ESM.tif]
